# Natural Convection Flow of a Nanofluid in an Inclined Square Enclosure Partially Filled with a Porous Medium

**DOI:** 10.1038/s41598-017-02241-x

**Published:** 2017-05-24

**Authors:** A. I. Alsabery, A. J. Chamkha, H. Saleh, I. Hashim

**Affiliations:** 1 Centre for Mathematical Sciences and Data Science, Faculty of Science & Technology, Universiti Kebangsaan Malaysia, 43600 UKM Bangi Selangor, Malaysia; 2 Department of Mechanical Engineering, Prince Mohammad Bin Fahd University, Al Khobar, 31952 Saudi Arabia; 3 Prince Sultan Endowment for Energy and the Environment, Prince Mohammad Bin Fahd University, P.O. Box 1664, Al-Khobar, 31952 Saudi Arabia

## Abstract

This work analyses free convection flow of a nanofluid in an inclined square enclosure consisting of a porous layer and a nanofluid layer using the finite difference methodology. Sinusoidal temperature boundary conditions are imposed on the two opposing vertical walls. Nanofluids with water as base and Ag or Cu or Al_2_O_3_ or TiO_2_ nanoparticles are considered for the problem. The related parameters of this study are the Darcy number, nanoparticle volume fraction, phase deviation, amplitude ratio, porous layer thickness and the inclination angle of the cavity. A comparison with previously published work is performed and the results are in good agreement. Detailed numerical data for the fluid flow and thermal distributions inside the square enclosure, and the Nusselt numbers are presented. The obtained results show that the heat transfer is considerably affected by the porous layer increment. Several nanoparticles depicted a diversity improvement on the convection heat transfer.

## Introduction

Studies about free convective fluid flow and heat transfer in porous media domains have received considerable attention over the past few years and their findings are gaining significant importance. This is due to their ability to resolve a wide range of environmental situations or industrial applications such as, geothermal systems, thermal insulation, filtration processes, ground water pollution, storage of nuclear waste, drying processes, solidification of castings, storage of liquefied gases, biofilm growth, fuel cells. The problem of dealing with the fluid motions in the clear region and the porous medium has been studied for many years. Beavers and Joseph^1^ presented the simple situation of the boundary conditions between a porous media and a homogeneous fluid. Poulikakos *et al*.^2^ considered high value of Rayleigh in free convection in a fluid overlaying a porous bed using the Darcy model. Meanwhile, Beckermann *et al*.^3^ studied free convective flow and heat transfer between a fluid layer and a porous layer inside a rectangular cavity. Free convective heat and mass transfer in solidification was studied by Beckermann *et al*.^4^. On the other hand, Chen and Chen^5^ investigated convective stability in a superposed fluid and porous layer when heated from below. Heat transfer and fluid flow through fibrous insulation was studied by Le Breton *et al*.^6^. Singh and Thorpe^7^ presented a comparative study of different models of free convection in a confined fluid and overlying porous layer. The problem with studying the solute exchange by convective within estuarine sediments had been considered by Webster *et al*.^8^. Goyeau *et al*.^9^ discussed the problem of using one- or two domain formulations for the conservation equations. Meanwhile, Gobin *et al*.^10^ analyzed the specified subclass of such problems where free convection occupies venue in a closed cavity with a partially-saturated porous medium. Nessrine *et al*.^11^ have applied the non-Darcy model to study the flow and heat transfer characteristics in a pipe saturated porous medium. Sui *et al*.^12^ used analytically the homotopy analysis method to study the convection heat transfer and boundary layer in a power-law fluid through a moving conveyor and inclined plate. Their results indicated that increasing the inclination angle clearly improved the rate of heat transfer.

Bhattacharya and Das^13^ considered numerically different values of Rayleigh and Nusselt numbers on natural convective flow within a square-shaped cavity. Thermal fluids are very important for heat transfer in many industrial applications. The poor thermal conductivity of classical heat transfer fluids like water and oils is the fundamental restriction in improving the rendering and the compactness of several engineering applications. A solid commonly has a greater conductivity than a fluid, for instance, the thermal conductivity of copper (Cu) is higher 700 times compared to that of water, while it is 3000 times greater than that of the engine oil. A novel mechanism for enhancing the heat transfer is by utilizing solid particles in the base fluid (i.e. nanofluids) in the area of sizes 10–50 nm^14^. Due to the small sizes and the large particular surface areas of the nanoparticles, nanofluids possess eminent characteristics like rising thermal conductivity, less blockage in the transit of the fluid flow, longer stabilization and homogeneity^15^. Consequently, nanofluids have an enormous area of possible enforcements like as in automotive, electronics, and nuclear applications where enhanced heat transfer and effective heat dissipation are desired. Ramiar *et al*.^16^ studied the influences of axial conduction and variable properties on conjugate heat transfer of a nanofluid in a microchannel. Sundar *et al*.^17^ investigated the enhancement of thermal conductivity and viscosity of nanodiamond-nickel nanocomposite nanofluids. Arani *et al*.^18^ investigated the free convection in a filled nanofluid square cavity. Chamkha and Ismael^19^ conducted a numerical study to solve the problem of differentially heated and partially layered vertical porous cavity filled with a nanofluid on free convection, for the first time by using the Darcy-Brinkman model. Sui *et al*.^20^ gave an experimental study of multilevel equivalent agglomeration model for heat conduction enhancement in nanofluids. Zaraki *et al*.^21^ applied theoretically the finite-difference method to investigate the effects of size, shape, the type of nanoparticles, the type of base fluid, the working temperature of free convective boundary layer heat, and the mass transfer of nanofluids. Lin *et al*.^22^ conducted a numerical study on magnetohydrodynamic non-Newtonian nanofluid flow and heat transfer within a finite thin film and with the effect of the heat generation. They used four various kinds of nanoparticles where they concluded that the local Nusselt number decreased by increasing the solid volume fraction. Hamid *et al*.^23^ used Buongiorno’s model to investigate Non-alignment stagnation-point flow of a nanofluid past a permeable stretching/shrinking sheet. Based on the Darcy law, Zhang *et al*.^24^ considered numerically the chemical effects on a boundary layer flow and heat transfer within a porous medium plate saturated with a fluid and three kinds of nanoparticles. Nevertheless, the study of free convective flow of a nanofluid in a square cavity partially consist with a porous medium based on Darcy model has not been undertaken yet.

Lately, the problems of free convection in enclosures for different temperature conditions were given enormous interest by diverse investigations. Sarris *et al*.^25^ examined the free convection in a closed cavity when the top wall has sinusoidal temperature condition whilst the remaining walls were kept insulated. Saeid and Yaacob^26^ studied the free convective heat transfer in an enclosure with variable hot left wall temperature and a constant cold right wall temperature. Bilgen and Yedder^27^ investigated the free convective heat transfer in a rectangular enclosure when the left vertical wall of the enclosure has a sinusoidal temperature distributions and the remaining walls were adiabatic. Deng and Chang^28^ discussed numerically the convective heat transfer in a rectangular-shaped cavity when the vertical walls heated using variable sinusoidal temperatures. Sathiyamoorthy and Chamkha^29–31^ investigated convection flow in an enclosure with linearly vertical heated walls. Bhuvaneswari *et al*.^32^ considered MHD convection in a square enclosure with sinusoidal temperature fields on both vertical walls. Chamkha *et al*.^33^ examined magnetohydrodynamics convection in a rectangular-shaped enclosure together with linearly concentrated and vertical heated walls. Cheong *et al*.^34^ carried out a study on the effects of aspect ratio on free convective in an inclined rectangular enclosure with sinusoidal on the vertical left wall. Kefayati *et al*.^35^ applied the Lattice Boltzmann methodology to study the magneto-convection flow of a nanofluid in a cavity. Ben-Cheikh *et al*.^36^ analyzed the free convection flow of a nanofluid in a square enclosure heated by a variable temperature field within the bottom horizontal wall. Bouhalleb and Abbassi^37^ employed the finite-volume element technique to solve the problem of free convective flow of a nanofluid in an inclined rectangular enclosure with a sinusoidal thermal condition on right vertical boundary. However, the subject of free convective fluid and heat flow in an enclosure partially saturated porous media with sinusoidally heating temperatures on the walls has not been considered yet. The goal of this work is to study the influence of Darcian natural convective flow of a nanofluid and heat transfer characteristics in an inclined square enclosure with a partly saturated porous layer using sinusoidal boundary conditions.

## Mathematical formulation

**Figure 1:**
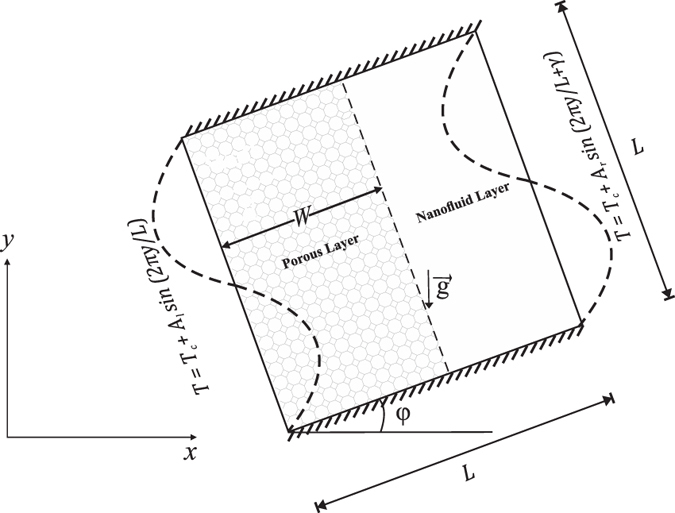
Physical model of free convection in an enclosure with the *x* and *y* coordinates and the inclination angle.

We consider the steady 2D free convective fluid and heat flow in a square-shaped enclosure with length *L*, the left cavity part filled with a porous layer *W*, while the remainder of the cavity (*L* − *W*) is filled with a nanofluid, as depicted in Fig. 1. The vertical walls of the enclosure are heated non-uniformly temperature (sinusoidal temperature), whilst the upper and bottom horizontal walls are adiabatic. The outer boundaries are considered to be impermeable, whilst the nanofluid layer boundaries are assumed to be permeable. The pores are filled with a fluid composed with Ag or Cu or Al_2_O_3_ or TiO_2_ nanoparticles in water as a base fluid. According to the Boussinesq approximation, the physical properties of the fluid are fixed but varies for the density. With the above assumptions, the conservation equations for mass, Darcy and energy equations for steady free convection for the porous and the nanofluid layers will be considered separately^38^

1 $$\frac{\partial {u}_{p}}{\partial x}+\frac{\partial {v}_{p}}{\partial y}=0,$$

2 $$\frac{\partial {u}_{p}}{\partial y}-\frac{\partial {v}_{p}}{\partial x}=\frac{{K}_{p}{\beta }_{p}\,g}{\nu }(\frac{\partial {T}_{p}}{\partial x}\,\cos \,\phi -\frac{\partial {T}_{p}}{\partial y}\,\sin \,\phi ),$$

3 $${u}_{p}\frac{\partial {T}_{p}}{\partial x}+{v}_{p}\frac{\partial {T}_{p}}{\partial y}=\alpha (\frac{{\partial }^{2}{T}_{p}}{\partial {x}^{2}}+\frac{{\partial }^{2}{T}_{p}}{\partial {y}^{2}}).$$

The conservation equations for mass, momentum and energy equation for the nanofluid layer are:

4 $$\frac{\partial {u}_{nf}}{\partial x}+\frac{\partial {v}_{nf}}{\partial y}=\mathrm{0,}$$

5 $$\begin{array}{rcl}{u}_{nf}\frac{\partial {u}_{nf}}{\partial x}+{v}_{nf}\frac{\partial {u}_{nf}}{\partial y} & = & -\frac{1}{{\rho }_{nf}}\frac{\partial {p}_{nf}}{\partial x}+{\nu }_{nf}(\frac{{\partial }^{2}{u}_{nf}}{\partial {x}^{2}}+\frac{{\partial }^{2}{u}_{nf}}{\partial {y}^{2}})\\  &  & +\,{\beta }_{nf}g({T}_{nf}-{T}_{c})\,\sin \,\phi ,\end{array}$$

6 $$\begin{array}{ccc}{u}_{nf}\frac{{\rm{\partial }}{v}_{nf}}{{\rm{\partial }}x}+{v}_{f}\frac{{\rm{\partial }}{v}_{nf}}{{\rm{\partial }}y} & = & -\frac{1}{{\rho }_{nf}}\frac{{\rm{\partial }}{p}_{nf}}{{\rm{\partial }}y}+{\nu }_{nf}(\frac{{{\rm{\partial }}}^{2}{v}_{nf}}{{\rm{\partial }}{x}^{2}}+\frac{{{\rm{\partial }}}^{2}{v}_{nf}}{{\rm{\partial }}{y}^{2}})\\  &  & +\,{\beta }_{nf}g({T}_{nf}-{T}_{c})\cos \phi ,\end{array}$$

7 $${u}_{nf}\frac{{\rm{\partial }}{T}_{nf}}{{\rm{\partial }}x}+{v}_{nf}\frac{{\rm{\partial }}{T}_{nf}}{{\rm{\partial }}y}={\alpha }_{nf}(\frac{{{\rm{\partial }}}^{2}{T}_{nf}}{{\rm{\partial }}{x}^{2}}+\frac{{{\rm{\partial }}}^{2}{T}_{nf}}{{\rm{\partial }}{y}^{2}}),$$

where *u* and *v* are the velocity components in the *x*-direction and *y*-direction, the subscripts *p*, *bf* and *nf* are related to solid matrix of the porous medium, the clear fluid in the porous medium (water) and the nanofluid saturated in the porous medium. *p* denotes the pressure, *ν* is the kinematic viscosity, *φ* is the inclination angle of the cavity, *T* represents the dimensional temperature, *K*_*p*_ is the permeability of the porous medium, *g* is the acceleration due to gravity, *ϕ* denotes the solid volume fraction, *k*_*nf*_ represents the effective nanofluid thermal conductivity, *ρ*_*nf*_ denotes the effective nanofluid density, *μ*_*nf*_ is the effective nanofluid dynamic viscosity and all these quantities are as defined below

8 $$\begin{array}{ccc}{\alpha }_{nf} & = & \frac{{k}_{nf}}{{(\rho {C}_{p})}_{nf}},\\ {\rho }_{nf} & = & (1-\varphi ){\rho }_{bf}+\varphi {\rho }_{sp},\\ \frac{{\mu }_{nf}}{{\mu }_{bf}} & = & \frac{1}{{(1-\varphi )}^{2.5}}.\end{array}$$

The heat capacitance of the nanofluids can be determined by

9 $${(\rho {C}_{p})}_{nf}=(1-\varphi )(\rho {C}_{p}{)}_{bf}+\varphi {(\rho {C}_{p})}_{sp}.$$

The nanofluids thermal expansion coefficient is given as

10 $${\beta }_{nf}=\mathrm{(1}-\varphi )(\beta {)}_{bf}+\varphi {\beta }_{sp},$$

11 $${(\rho \beta )}_{nf}=(1-\varphi )(\rho \beta {)}_{bf}+\varphi {(\rho \beta )}_{sp}.$$

The nanofluid thermal conductivity according to Maxwell-Garnett’s (MG) model is given below:

12 $$\frac{{k}_{nf}}{{k}_{bf}}=\frac{{k}_{sp}+2{k}_{bf}-2\varphi ({k}_{bf}-{k}_{sp})}{{k}_{sp}+2{k}_{bf}+\varphi ({k}_{bf}-{k}_{sp})}.$$

In terms of the stream function *ψ* and the vorticity *ω*, which are defined in usual way as:

13 $$u=\frac{{\rm{\partial }}\psi }{{\rm{\partial }}y},v=-\,\frac{{\rm{\partial }}\psi }{{\rm{\partial }}x},$$

14 $$\omega =\frac{{\rm{\partial }}v}{{\rm{\partial }}x}-\frac{{\rm{\partial }}u}{{\rm{\partial }}y},$$

and also introduce the following non-dimensional variables:

15 $$X=\frac{x}{L},Y=\frac{y}{L},{\rm{\Omega }}=\frac{\omega {L}^{2}}{{\alpha }_{bf}},{\rm{\Psi }}=\frac{\psi }{{\alpha }_{bf}},{\theta }_{nf}=\frac{{T}_{nf}-{T}_{c}}{{T}_{h}-{T}_{c}},{\theta }_{p}=\frac{{T}_{p}-{T}_{c}}{{T}_{h}-{T}_{c}}.$$

The dimensionless governing equations for the porous layer are:

16 $$\frac{{{\rm{\partial }}}^{2}{{\rm{\Psi }}}_{p}}{{\rm{\partial }}{X}^{2}}+\frac{{{\rm{\partial }}}^{2}{{\rm{\Psi }}}_{p}}{{\rm{\partial }}{Y}^{2}}=R{a}_{bf}Da(\frac{{\rm{\partial }}{\theta }_{p}}{{\rm{\partial }}X}\cos \phi -\frac{{\rm{\partial }}{\theta }_{p}}{{\rm{\partial }}Y}\sin \phi ),$$

17 $$\frac{\partial {{\rm{\Psi }}}_{p}}{\partial X}\frac{\partial {\theta }_{p}}{\partial Y}-\frac{\partial {{\rm{\Psi }}}_{p}}{\partial Y}\frac{\partial {\theta }_{p}}{\partial X}=\frac{{k}_{p}}{{k}_{nf}}(\frac{{\partial }^{2}{\theta }_{p}}{\partial {X}^{2}}+\frac{{\partial }^{2}{\theta }_{p}}{\partial {Y}^{2}}).$$

The dimensionless governing equations for the nanofluid layer can be written as:

18 $$\frac{{\partial }^{2}{{\rm{\Psi }}}_{nf}}{\partial {X}^{2}}+\frac{{\partial }^{2}{{\rm{\Psi }}}_{nf}}{\partial {Y}^{2}}=-\,{{\rm{\Omega }}}_{nf},$$

19 $$\begin{array}{ccc}\frac{{\rm{\partial }}{{\rm{\Psi }}}_{nf}}{{\rm{\partial }}Y}\frac{{\rm{\partial }}{{\rm{\Omega }}}_{nf}}{{\rm{\partial }}X}-\frac{{\rm{\partial }}{{\rm{\Psi }}}_{nf}}{{\rm{\partial }}X}\frac{{\rm{\partial }}{{\rm{\Omega }}}_{nf}}{{\rm{\partial }}Y} & = & \frac{{\mu }_{nf}}{{\mu }_{bf}}[\frac{P{r}_{bf}}{(1-\varphi )+\varphi \frac{{\rho }_{sp}}{{\rho }_{bf}}}](\frac{{{\rm{\partial }}}^{2}{{\rm{\Omega }}}_{nf}}{{\rm{\partial }}{X}^{2}}+\frac{{{\rm{\partial }}}^{2}{{\rm{\Omega }}}_{nf}}{{\rm{\partial }}{Y}^{2}})\\  &  & +\,\frac{{\beta }_{nf}}{{\beta }_{bf}}R{a}_{bf}P{r}_{bf}(\frac{{\rm{\partial }}{\theta }_{nf}}{{\rm{\partial }}X}\cos \phi -\frac{{\rm{\partial }}{\theta }_{nf}}{{\rm{\partial }}Y}\sin \phi ),\end{array}$$

20 $$\frac{{\rm{\partial }}{{\rm{\Psi }}}_{nf}}{{\rm{\partial }}Y}\frac{{\rm{\partial }}{\theta }_{nf}}{{\rm{\partial }}X}-\frac{{\rm{\partial }}{{\rm{\Psi }}}_{nf}}{{\rm{\partial }}X}\frac{{\rm{\partial }}{\theta }_{nf}}{{\rm{\partial }}Y}=\frac{{(\rho {C}_{p})}_{bf}}{{(\rho {C}_{p})}_{nf}}\frac{{k}_{nf}}{{k}_{bf}}(\frac{{{\rm{\partial }}}^{2}{\theta }_{nf}}{{\rm{\partial }}{X}^{2}}+\frac{{{\rm{\partial }}}^{2}{\theta }_{nf}}{{\rm{\partial }}{Y}^{2}}),$$

where $R{a}_{bf}=g{\rho }_{bf}{\beta }_{bf}({T}_{h}-{T}_{c}){L}^{3}/({\mu }_{bf}{\alpha }_{bf})$ represents Rayleigh number of water, *Da* = *K*/*L* ^2^ is the Darcy number for porous layer and *Pr* = *ν*_*f*_/*α*_*f*_ denotes Prandtl number of water. The dimensionless boundary conditions for solving equations (16)–(20) are:

21 $${\rm{\Omega }}=-\,\frac{{{\rm{\partial }}}^{2}{\rm{\Psi }}}{{\rm{\partial }}{X}^{2}},{\theta }_{l}={\theta }_{p}=\sin (2\pi Y){\rm{a}}{\rm{t}}X=0,$$

22 $${\rm{\Omega }}=-\,\frac{{{\rm{\partial }}}^{2}{\rm{\Psi }}}{{\rm{\partial }}{X}^{2}},{\theta }_{r}={\theta }_{nf}=\varepsilon \sin (2\pi Y+\gamma ){\rm{a}}{\rm{t}}X=1,$$

23 $${\rm{\Omega }}=-\,\frac{{{\rm{\partial }}}^{2}{\rm{\Psi }}}{{\rm{\partial }}{Y}^{2}},\frac{{\rm{\partial }}{\theta }_{p}}{{\rm{\partial }}Y}=\frac{{\rm{\partial }}{\theta }_{nf}}{{\rm{\partial }}Y}=0{\rm{a}}{\rm{t}}Y=0,Y=1,$$

and at the interface by using the matching conditions proposed by Beavers and Joseph^1^

24 $$\begin{array}{ccc}\frac{{\rm{\partial }}{{\rm{\Psi }}}^{+}}{{\rm{\partial }}X} & = & \frac{{\rm{\partial }}{{\rm{\Psi }}}^{-}}{{\rm{\partial }}X},\\ \frac{{{\rm{\partial }}}^{2}{{\rm{\Psi }}}^{+}}{{\rm{\partial }}{X}^{2}} & = & \bar{\alpha }(\frac{{\rm{\partial }}{{\rm{\Psi }}}^{+}}{{\rm{\partial }}X}-\frac{{\rm{\partial }}{{\rm{\Psi }}}^{-}}{{\rm{\partial }}X})/\sqrt{Da},\\ {\theta |}_{X={s}^{+}} & = & {\theta |}_{X={s}^{-}},\\ {k}_{nf}\frac{{\rm{\partial }}{\theta }_{nf}}{{\rm{\partial }}X}{s}^{+} & = & {k}_{p}\frac{{\rm{\partial }}{\theta }_{p}}{{\rm{\partial }}X}{s}^{-},\\ {{\rm{\Omega }}}^{+} & = & -\frac{{{\rm{\partial }}}^{2}{{\rm{\Psi }}}^{+}}{{\rm{\partial }}{X}^{2}}+\frac{{{\rm{\partial }}}^{2}{{\rm{\Psi }}}^{+}}{{\rm{\partial }}{Y}^{2}}.\end{array}$$

In our study the value of $\bar{\alpha }$ fix at 1, and the subscripts + and − indicate that the respective quantities are evaluated while approaching the interface from the nanofluid and porous layers respectively.

The local Nusselt number for both vertical walls, which are defined, respectively, by

25 $$N{u}_{l}=-\,{(\frac{\partial {\theta }_{p}}{\partial X})}_{X=0},$$

26 $$N{u}_{r}=-\,{(\frac{\partial {\theta }_{nf}}{\partial X})}_{X=1}.$$

The fluid will gain heat from the half-heated vertical sidewall which yields *Nu* > 0. However, the fluid tends to lose the thermal heating from the half-cold vertical sidewall and hence *Nu* < 0. Adding the average Nusselt numbers of the two heated parts of the vertical boundaries will give the heat transfer rate of the cavity, given as^39^:

27 $${\overline{Nu}}_{nf}=(\frac{{k}_{nf}}{{k}_{bf}})[{\int }_{{\rm{heating}}{\rm{half}}}N{u}_{l}\,{\rm{d}}Y+{\int }_{{\rm{heating}}{\rm{half}}}N{u}_{r}\,{\rm{d}}Y].$$

## Numerical Method and Validation

**Figure 2:**
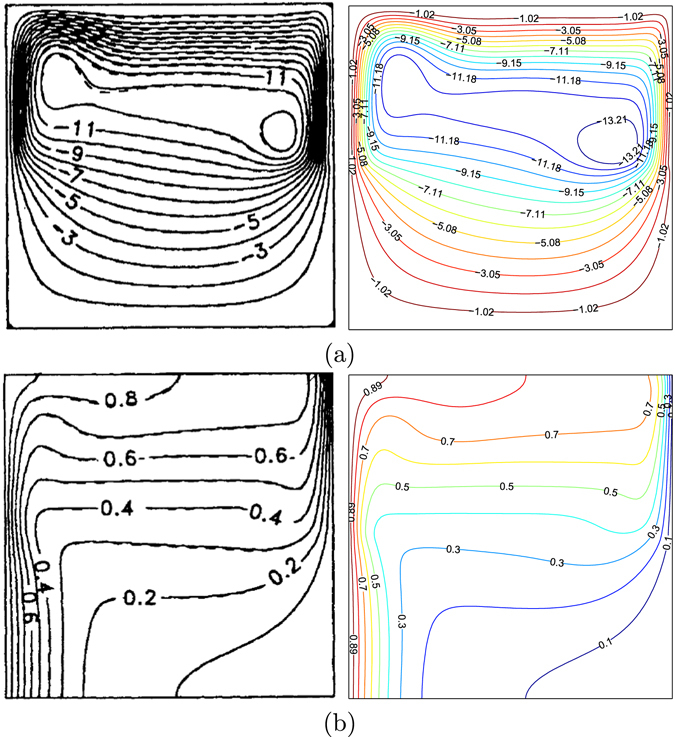
Streamlines (**a**), Singh and Thorpe^7^ (left), present study (right), isotherms (**b**), Singh and Thorpe^7^ (left), present study (right) for *Ra* = 10^6^, *Da* = 10^−5^, *ϕ* = 0, *S* = 0.5, *φ* = 0 and Pr = 0.71.

**Figure 3:**
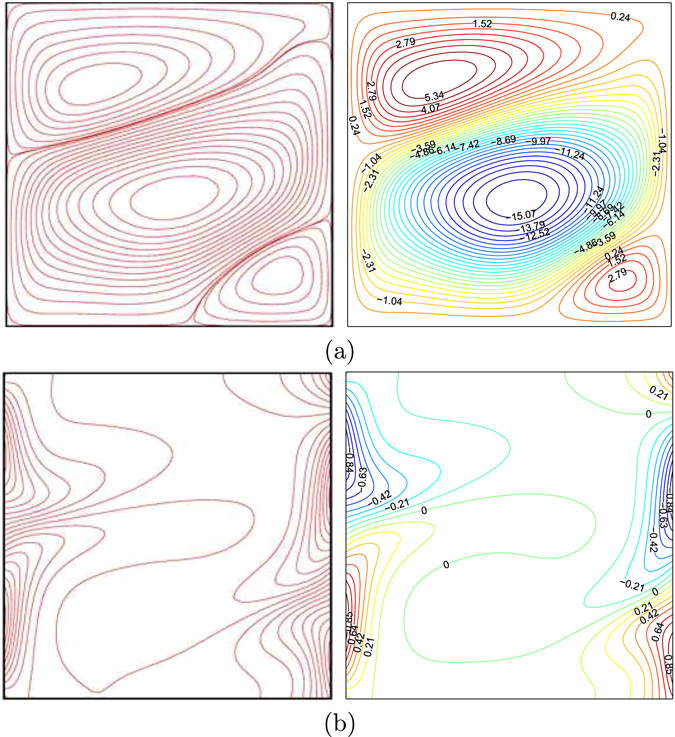
Streamlines (**a**), Deng and Chang^28^ (left), present study (right), isotherms (**b**), Deng and Chang^28^ (left), present study (right) for *ϕ* = 0, *φ* = 0 and Pr = 0.71.

In the current work, the Finite Difference (FD) methodology is utilized for solving the dimensionless governing equations (16)–(20) subject to the corresponding boundary conditions (21)–(24) depending on the Alternating Direction Implicit (ADI) method and the Tri-Diagonal Matrix Algorithm (TDMA). The numerical results of the current study compare to the results presented by Singh and Thorpe^7^ exhibits a very good validation by calculating the values of $\overline{Nu}$ with various values of the Rayleigh number and fluid layer thickness (*D*) with the fixed temperature for the vertical walls at *Da* = 10^−4^, *ϕ* = 0 and *φ* = 0 as shown in Table 1. Apart from that, we also compared the present figures with the ones provided by Singh and Thorpe^7^ for *Ra* = 10^6^, *Da* = 10^−5^, *ϕ* = 0, *S* = 0.5 and *φ* = 0 as depicted in Fig. 2. In addition, we have compared the current figures with the ones presented by by Deng and Chang^28^ for pure fluid (Pr = 0.71) as shown in Fig. 3. Figure 3 demonstrates the results of this paper with the related figures presented by Deng and Chang^28^ for sinusoidal boundary conditions on both sidewalls at *Ra* = 10^5^, $\gamma =\tfrac{\pi }{4}$, *ε* = 1, *ϕ* = 0, *S* = 0 and *φ* = 0. These data results supply the dependability to gauge the precision of the current numerical methodology.

**Table 1:** Comparison of $\overline{{\boldsymbol{Nu}}}$ for Darcy model for porous media with horizontal partition at a constant left and right wall temperature for *Da* = 10^−4^ and *ϕ* = 0.

*Ra*	*D*	Singh and Thorpe^7^	Present
10^4^	0.3	1.061	1.063
10^4^	0.5	1.548	1.550
10^5^	0.3	2.190	2.191
10^5^	0.5	3.376	3.377
10^6^	0.3	5.432	5.432
10^6^	0.5	6.950	6.949

## Results and Discussion

We provide in this part a numerical results for the streamlines of the porous/nanofluid-layers and isotherms of the porous/nanofluid-layers with various values of the Darcy number (10^−5^ ≤ *Da* ≤ 10^−3^), nanoparticle volume fraction (0 ≤ *ϕ* ≤ 0.2), phase deviation, (0 ≤ *γ* ≤ *π*), amplitude ratio, (0 ≤ *ε* ≤ 1), porous layer thickness (0.1 ≤ *S* ≤ 0.9), inclination angle of the cavity (0° ≤ *φ* ≤ 90°), Rayleigh number (*Ra*_*bf*_ = 10^4^, 10^5^) and the Prandtl number (*Pr*_*bf*_ = 6.2). The values of the average Nusselt number are calculated for various values of *φ* and *S*. Table 2 lists the water base fluid (*Pr*_*bf*_ = 6.2) with the thermo-physical properties of the considered nanoparticles.

**Table 2:** Thermo-physical properties of water with Ag, Cu, Al_2_O_3_ and TiO_2_.

Physical properties	Water	Ag	Cu	Al_2_O_3_	TiO_2_
*C*_*p*_ (J/kgK)	4179	235	383	765	686.2
*ρ* (kg/m^3^)	997.1	10500	8954	3600	4250
*k* (Wm^−1^ K^−1^)	0.6	429	400	46	8.954
*β* × 10^−5^ (1/K)	21	5.4	1.67	0.63	2.4

**Figure 4:**
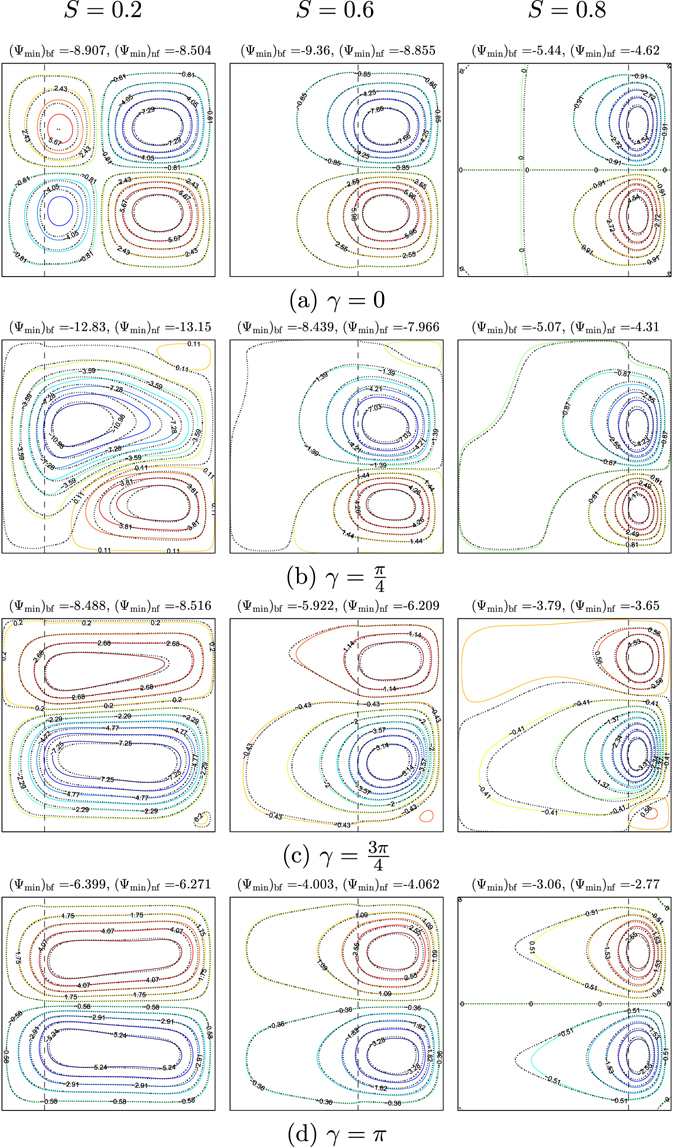
Streamlines evolution by *γ* and *S* for *Ra*_*bf*_ = 10^5^, *Da* = 10^−4^, *ε* = 1, *φ* = 0°, *ϕ* = 0 (solid lines) and *ϕ* = 0.1 (dashed lines).

**Figure 5:**
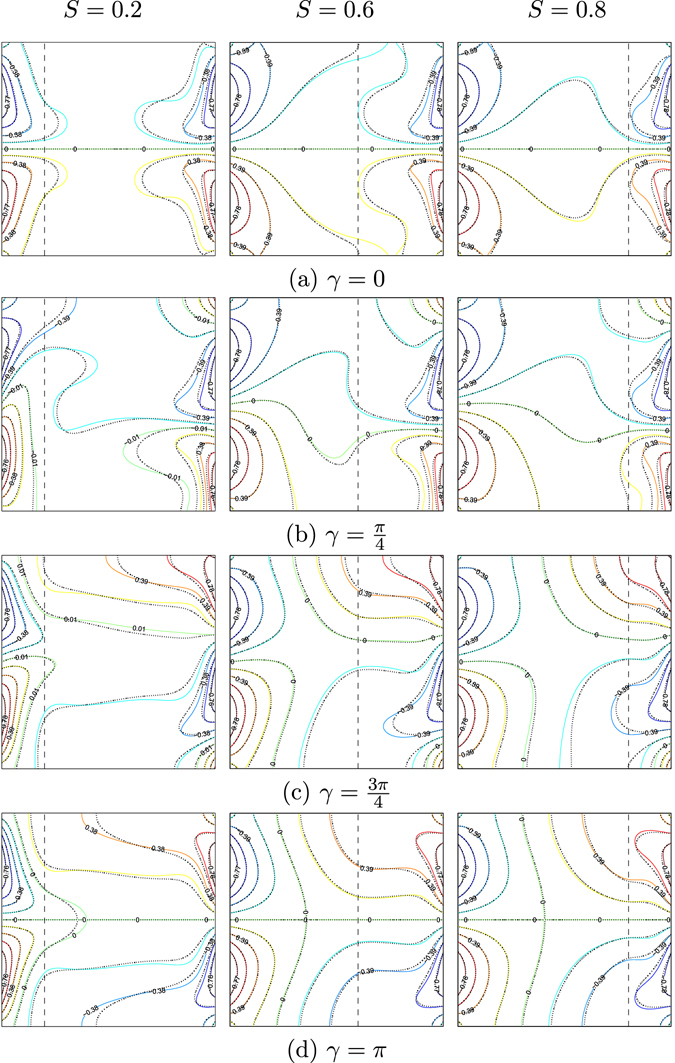
Isotherms evolution by *γ* and *S* for *Ra*_*bf*_ = 10^5^, *Da* = 10^−4^, *ε* = 1, *φ* = 0°, *ϕ* = 0 (solid lines) and *ϕ* = 0.1 (dashed lines).

Figures 4 and 5 exhibit the influences of several values of *γ* on the streamlines and isotherms for various values of the porous layer thickness (*S* = 0.2, 0.6 and 0.8) for water–Cu at *Ra*_*bf*_ = 10^5^, *Da* = 10^−4^, *ε* = 1 and *φ* = 0°. Heating the vertical walls of the enclosure using similar temperature profiles (*γ* = 0) is clearly affected the flow behavior. At a smaller porous layer thickness (*S* = 0.2), the flow within the cavity appears with two symmetric cells next the left wall (one in the anticlockwise direction and the other in the clockwise direction) and two symmetric cells near to the right vertical wall. Where the positive sign of Ψ denotes anti-clockwise fluid heat flow, the negative sign of Ψ designates the clockwise fluid heat flow. When the streamlines circulated in the form of vortices in the clockwise direction (negative signs of Ψ), the strength of the flow circulation was denoted as Ψ_min_. Adding 0.1 of Cu nanoparticles does not help the flow motion due to the non-uniform heating on the walls. The reduction of the strength of the flow circulation appears clearly with the application of the nanofluid (see Ψ_min_ values). The intensity of the streamlines occurs strongly into the nanofluid layer with two symmetric circulation cells with the porous layer increment. The intensity of the isotherms pattern increases with the increase of the porous layer thickness. Affected by the resistance of the porous layer hydrodynamics, the strength of the flow circulation decreases with the increase of the porous layer to the maximum value (*S* = 0.8), as depicted in Figs 4(a) and 5(a). As the phase deviation increases ($\gamma =\tfrac{\pi }{4}$), the flow structure is significantly enhanced. The clockwise circulation streamlines cell is almost seized into the cavity, while the anti-clockwise circulation streamlines cell appears close to the bottom wall. The strength of the flow circulation increases with phase deviation increment, due to the least possible thickness of the porous layer. The strength of the flow circulation increases with the addition of Cu nanoparticles (see Ψ_min_ values). Increasing the phase deviation to *π* might slow down the flow with two similar streamlines cells for all *S* values. The intensity of the isotherms pattern tends to be more spread out within the cavity, as shown in Figs 4(d) and 5(d).

**Figure 6:**
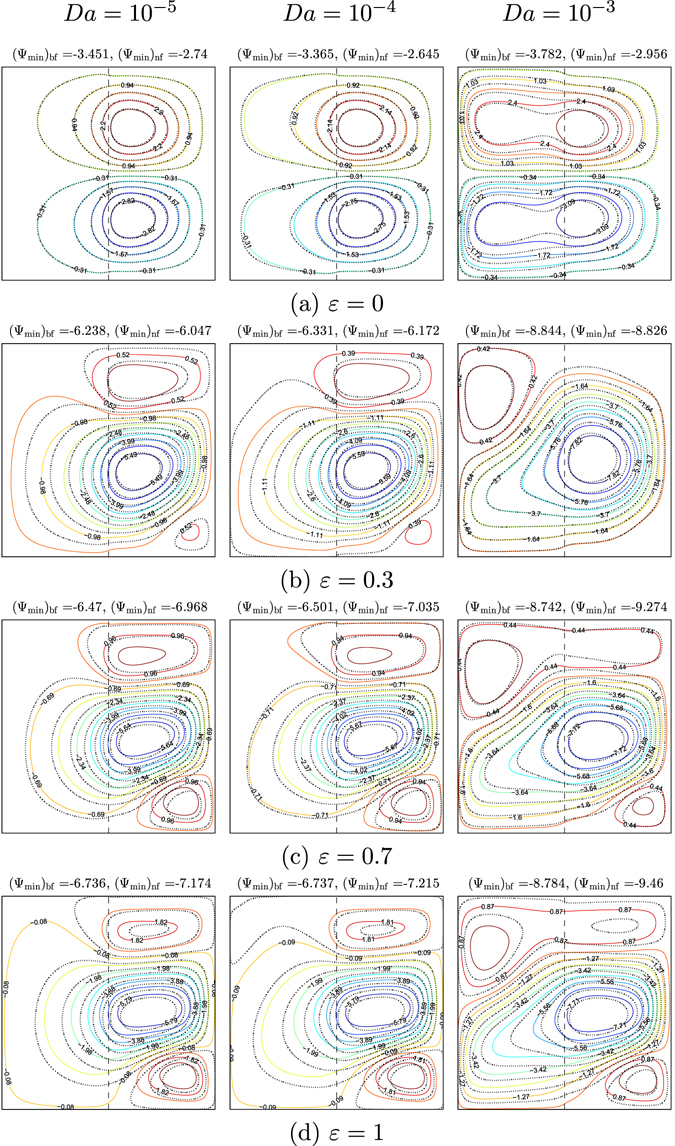
Streamlines evolution by *ε* and *Da* for *Ra*_*bf*_ = 10^5^, $\gamma =\tfrac{\pi }{2}$, *S* = 0.5, *φ* = 0°, *ϕ* = 0 (solid lines) and *ϕ* = 0.1 (dashed lines).

**Figure 7:**
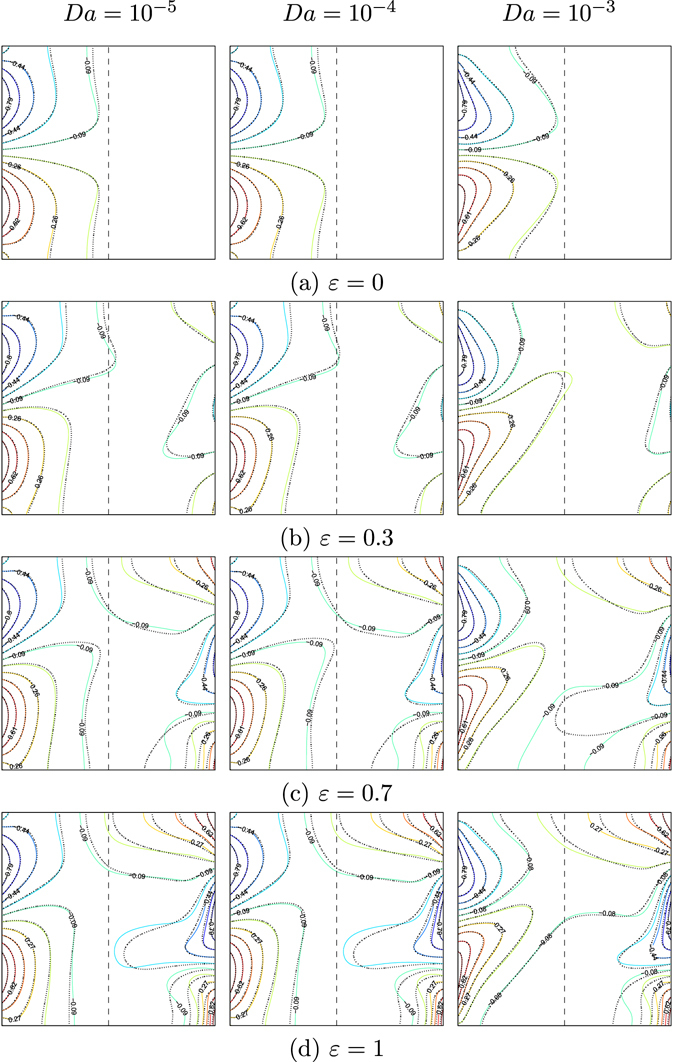
Isotherms evolution by *ε* and *Da* for *Ra*_*bf*_ = 10^5^, $\gamma =\tfrac{\pi }{2}$, *S* = 0.5, *φ* = 0°, *ϕ* = 0 (solid lines) and *ϕ* = 0.1 (dashed lines).

Figures 6 and 7 illustrate the effects of different values of the amplitude ratio on the streamlines and isotherms for various values of Darcy number (*Da* = 10^−5^, 10^−4^ and 10^−3^) for water–Cu at *Ra*_*bf*_ = 10^5^, $\gamma =\tfrac{\pi }{2}$, *S* = 0.5 and *φ* = 0°. The effects of constant temperature (*ε* = 0) on the flow motion and the temperature profiles are presented in Figs 6(a) and 7(a). The streamlines in the enclosure show two symmetric cells at the top and bottom parts of the nanofluid layer in the enclosure. This is due to the absence of *ε* on the right vertical wall. We observed strong effects on the flow behavior by increasing the Darcy number, due to the buoyancy force or convection intensity increment. The two streamlines in the symmetric cells tend to expand with the movement towards the porous layer. This behavior appears clearly in the nanofluid compared to that of pure fluid. Consequently, the strength of the flow circulation increases along with the alteration of the Darcy number. Affected by the constant temperature on the right wall, the isotherms pattern distributions appear closer near the left wall of the cavity. The flow behavior is clearly affected when the low value of amplitude ratio (*ε* = 0.3) and Darcy number are applied as the streamlines appointed three various cells within the cavity. At the center of the cavity, the major clockwise circulation cell appears clearly, while the two secondary anti-clockwise circulation cells take place closer to the adiabatic walls. When *Da* = 10^−3^, the strength of the flow circulation increases with the addition of the nanoparticles due to the kinetic energy increment. Distinctly, the distortion of the flow structure at a higher amplitude ratio (1) is due to the non-uniform temperature on the vertical walls. The intensity and the amount of the isotherms patterns increased along the left and right walls owing to the enhancement in the amplitude ratio, as presented in Figs 6(d) and 7(d).

**Figure 8:**
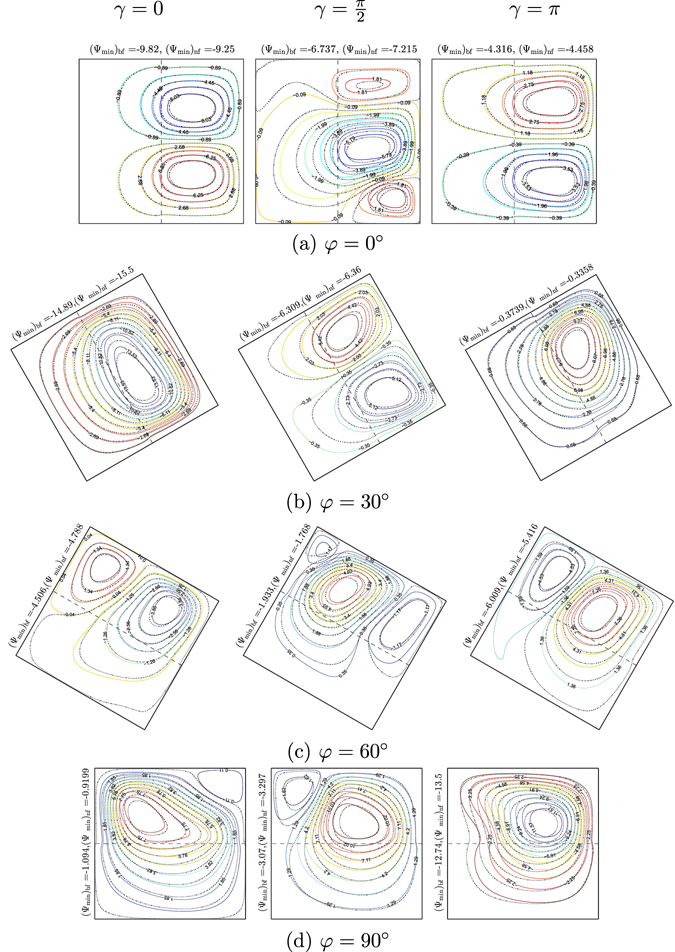
Streamlines evolution by *φ* and *γ* for *Ra*_*bf*_ = 10^5^, *Da* = 10^−4^, *ε* = 1, *S* = 0.5, *ϕ* = 0 (solid lines) and *ϕ* = 0.1 (dashed lines).

**Figure 9:**
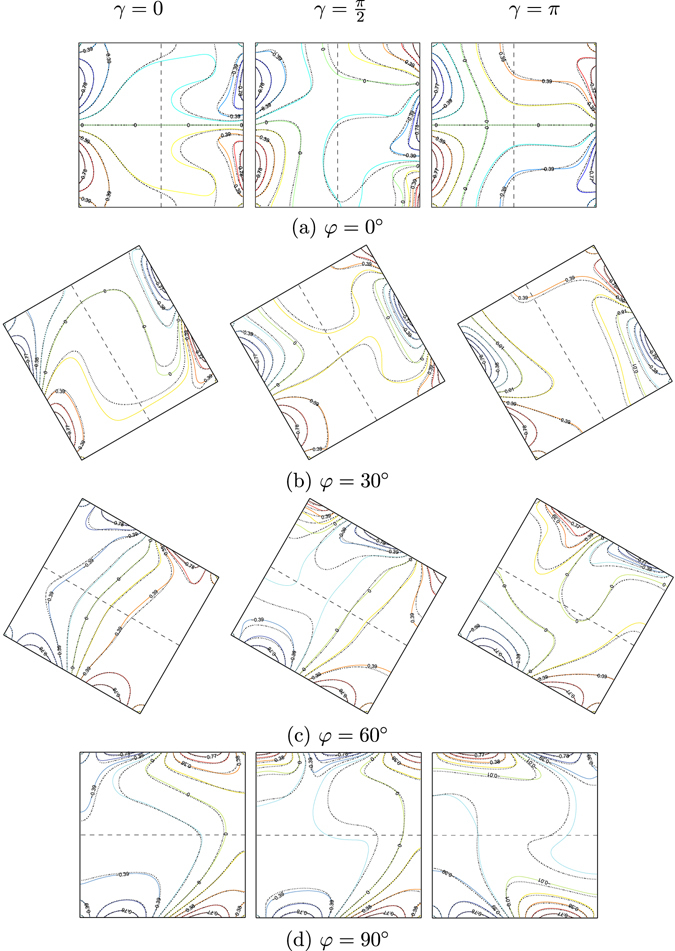
Isotherms evolution by *φ* and *γ* for *Ra*_*bf*_ = 10^5^, *Da* = 10^−4^, *ε* = 1, *S* = 0.5, *ϕ* = 0 (solid lines) and *ϕ* = 0.1 (dashed lines).

Figures 8 and 9 demonstrate the effects of inclination angle of the enclosure on the streamlines and isotherms for different values of phase deviation (*γ* = 0, $\gamma =\tfrac{\pi }{2}$ and *γ* = *π*) for water–Cu at *Ra*_*bf*_ = 10^5^, *Da* = 10^−4^, *ε* = 1 and *S* = 0.5. The flow structure and the temperature profiles in the absence of the inclination angle (*φ* = 0°), are illustrated in Figs 8(a) and 9(a). Similar to the previous results, the flow within the cavity appears with two symmetric cells at the upper and lower segments of the nanofluid layer. Increasing the phase deviation value tends to alter the flow behavior which is affected by the non-uniform temperature profile on the vertical walls of the cavity. The streamlines allocate the flow composition of three various cells located at the cavity center and close to the adiabatic walls. As the phase deviation enhances, the strength of flow circulation rises with the addition of the nanofluid (see Ψ_min_ values). The isotherm patterns are clearly influenced by the phase deviation increment, due to the heat transfer improvement. We observe significant changes in the flow structure by applying lower inclination angle value (*φ* = 30°), the streamlines appear as a clockwise rotating cell within the nanofluid layer. In other words, imposing a lower inclination angle value forces the streamlines to appear as a singular rotating cell which is similar to the constant temperature distribution. At a higher phase deviation value (*γ* = *π*), the flow behavior is significantly affected. The circulation of the streamlines is characterized by a singular anti-clockwise rotating cell. Increasing the inclination angle to the higher value (*φ* = 90°) leads to the redistribution of the streamlines and the isotherm patterns. The reason to this behavior is due to the gravity force pushing the fluid flow and the changes in the flow direction appear similar to the constant heating from below, as shown in Figs 8(d) and 9(d).

**Figure 10:**
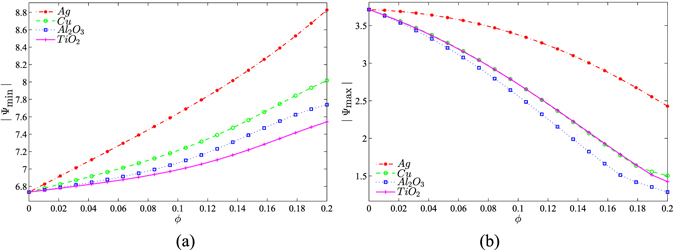
Values of the absolute (**a**) minimum and (**b**) maximum values of Ψ with *ϕ* for different nanoparticles at *Ra*_*bf*_ = 10^5^, *Da* = 10^−4^, $\gamma =\tfrac{\pi }{2}$, *ε* = 1, *S* = 0.5 and *φ* = 0°.

Figure 10 depicts the effect of various nanoparticles absolute lower and upper values of strength of the streamlines with nanoparticle volume fractions for *Ra*_*bf*_ = 10^5^, *Da* = 10^−4^, $\gamma =\tfrac{\pi }{2}$, *ε* = 1, *S* = 0.5 and *φ* = 0°. Figure 10(a) clearly indicates that the absolute lower value of the strength of the flow circulation enhances with the addendum of the nanofluid. This is owing to the increment in the viscosity forces and the inertial force. The strong enhancement of the strength of the streamlines obtained with the Ag nanoparticles is owing to the greater thermal conductivity of Ag. In addition, this behavior leads to appear visibly with a greater concentration of nanoparticles (*ϕ* ≥ 0.1). However, we observe different effects on the absolute upper values of the strength of the flow circulation of the nanoparticles volume fraction increment, as presented in Fig. 10(b). This figure also shows that the strength of the flow circulation significantly decreases with the addendum of nanofluid for all nanoparticles kinds.

**Figure 11:**
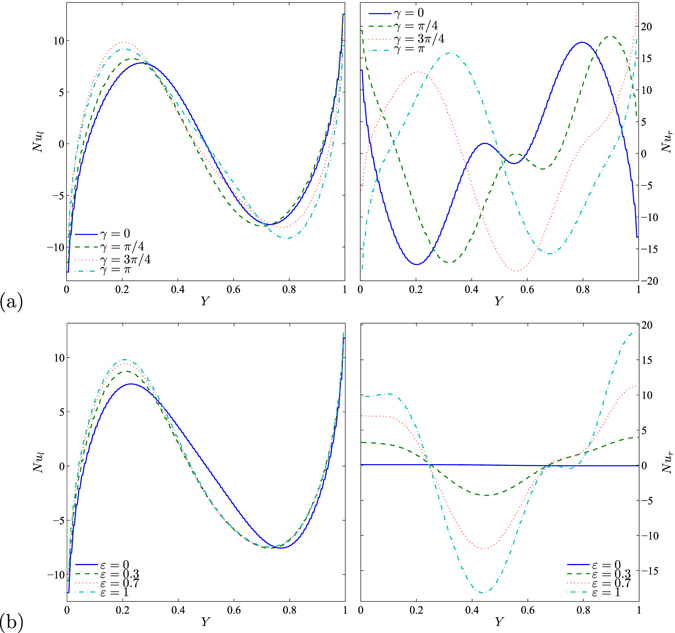
Values of *Nu* interfaces with *Y* for (**a**) different *γ* and (**b**) different *ε* at *Ra*_*bf*_ = 10^5^, *Da* = 10^−4^, *ϕ* = 0.1, *S* = 0.5 and *φ* = 0°.

Figure 11 shows the effects of various values of the amplitude ratio and phase deviation, respectively on *Nu* and along the *Y* coordinates for water–Cu, *Ra*_*bf*_ = 10^5^, *Da* = 10^−4^, *ϕ* = 0.1, *S* = 0.5 and *φ* = 0°. The effects of different values of phase deviation respectively for vertical left and right sides on the local Nusselt number and along the *Y* coordinates is depicted in Fig. 11(a). Obviously, the heat transfer enhancement on the right wall appears stronger than that on left wall due to the change in the phase deviation. The curved lines of the local Nusselt number on the left vertical wall are enhanced weakly by the changes in the phase deviation. Increasing the phase deviation from 0 to *π* caused the heating domain on the right wall to transfer to the top, while the cooling domain tends to transfer to the bottom which forces the local Nusselt number to appear with sinusoidal form. Figure 11(b) illustrates the effects of various values of amplitude ratio for the left and right vertical walls respectively on *Nu* and along the *Y* coordinates. At the right vertical wall there is no changing on the heat transfer with the absence of *ε*. Increasing the amplitude ratio value leads to a remarkable enhancement in the heat transfer. Furthermore, a higher amplitude ratio (*ε* = 1) substantially promotes the heat transfer in which the higher value of the local average Nusselt number can be obtained.

**Figure 12:**
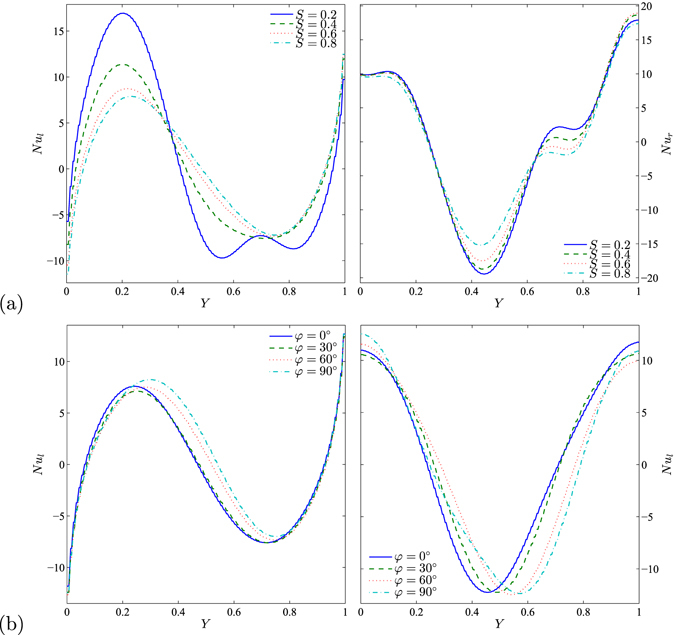
Values of *Nu* interfaces with *Y* for (**a**) different *S* and (**b**) different *φ* at *Ra*_*bf*_ = 10^5^, *Da* = 10^−4^, $\gamma =\tfrac{\pi }{2}$, *ε* = 1 and *ϕ* = 0.1.

Figure 12 clearly demonstrates the effects of various values of porous layer thickness and inclination angle respectively on the local Nusselt number and along the *Y* coordinates for water–Cu, *Ra*_*bf*_ = 10^5^, *Da* = 10^−4^, $\gamma =\tfrac{\pi }{2}$, *ε* = 1 and *ϕ* = 0.1. Figure 12(a) shows the effects of various values of porous layer thickness for vertical left and right sides on *Nu* and along the *Y* coordinates. It can be clearly observed from the Nusselt number curves that the convection heat transfer is clearly increased by heating the lower part and cooling the upper part of the left wall. In other words, the heat transfer is enhanced with the heating of the lower half of the left vertical wall, while cooling the upper half of the wall tends to decrease the heat transfer enhancement. In the right vertical wall, from the curves of the local Nusselt numbers, we observe the significant effect of heat transfer through temperature distribution. Figure 12(b) presents the effects of various values of the inclination angle for the vertical walls on *Nu* and along the *Y* coordinates for water–Cu, *Ra*_*bf*_ = 10^4^, *Da* = 10^−4^, $\gamma =\tfrac{\pi }{2}$, *ε* = 1, *ϕ* = 0.1 and *S* = 0.5. Due to the non-uniform temperature on the vertical walls, the enhancement of the heat distribution causes the heating domain on the right wall to transfer to the top, while the cooling domain tends to transfer to the bottom for all *φ* values. On the right wall, the Nusselt number curves tend to form a *V* shape for *φ* values affected by the velocity variation.

**Figure 13:**
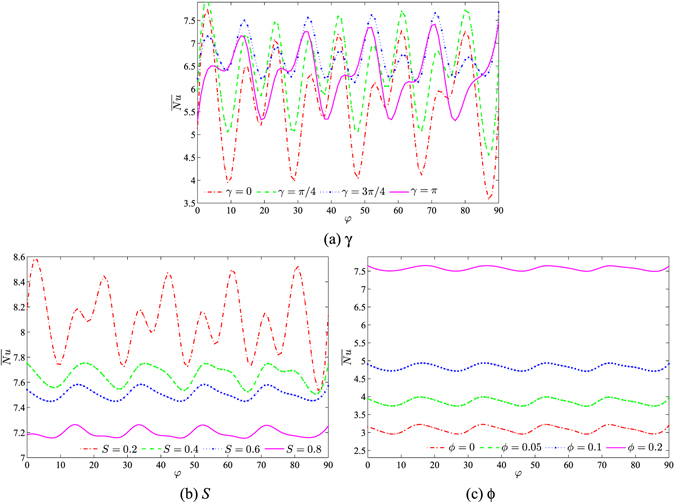
Values of $\overline{Nu}$ interfaces with *φ* for (**a**) different *γ*, (**b**) different *S* and (**c**) different *ϕ* at *Ra*_*bf*_ = 10^5^, *Da* = 10^−4^ and *ε* = 1.

The effects of various parameter values on $\overline{Nu}$ with the inclination angle of the cavity are clearly displayed in Fig. 13, for water–Cu, *Ra*_*bf*_ = 10^5^, *Da* = 10^−4^ and *ε* = 1. The convection heat transfer is significantly influenced by the inclination angle increment affected by the velocity variation. The strong enhancement of the rate of heat transfer is obtained by making changes to the phase deviation. The increment and the reduction of the average Nusselt number are indicated by the fixed inclination angle. The minimum reduction in the convection heat transfer rate appears with the absence of the phase deviation (*γ* = 0), while the maximum increment occurs when the value of *γ* is equal to $\tfrac{\pi }{4}$. In addition, this graph shows that the best heat transfer enhancement is obtained with the smallest porous layer thickness (*S* = 0.2). Furthermore, the higher concentration of solid volume fraction (*ϕ* = 0.2) together with the smallest porous layer thickness are able to influence the heat transfer distribution which results in the higher average Nusselt number.

**Figure 14:**
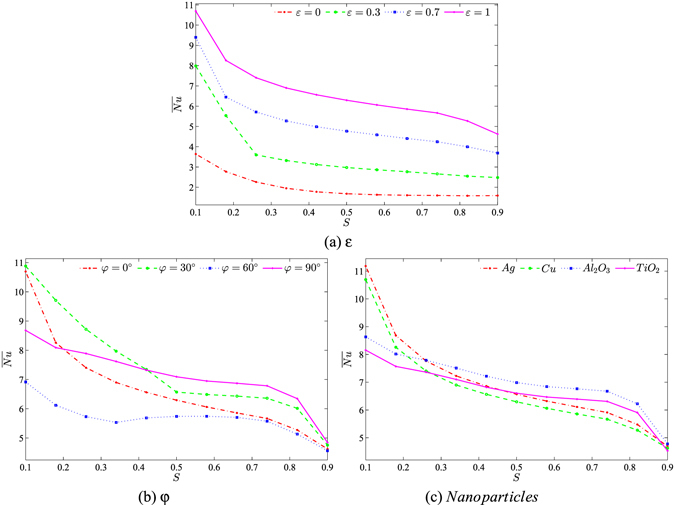
Values of $\overline{Nu}$ interfaces with *S* for (**a**) different *ε*, (**b**) different *φ* and (**c**) different *sp* at *Ra*_*bf*_ = 10^5^, *Da* = 10^−4^, $\gamma =\tfrac{\pi }{2}$ and *ϕ* = 0.1.

Figure 14 illustrates the effects of various parameter values on the average Nusselt number with the porous layer thickness for water–Cu, *Ra*_*bf*_ = 10^5^, *Da* = 10^−4^, $\gamma =\tfrac{\pi }{2}$ and *ϕ* = 0.1. The convection heat transfer is systematically reduced with an increment in the porous layer thickness due to the resistance of the porous layer hydrodynamics. As the amplitude ratio increases, the heat transfer rate is enhanced and this is caused by the non-uniform temperature distribution. In addition, higher amplitude ratio (*ε* = 1) strongly enhances the heat transfer rate which leads to the maximum average Nusselt number values. This figure also indicates that the heat transfer rate assumes various behaviors with increment of the inclination angle due to changes in the velocity. Figure 14(c) clearly depicts the effect of various values of the nanoparticles on $\overline{Nu}$ with the porous layer thickness for *Ra*_*bf*_ = 10^5^, *Da* = 10^−4^, $\gamma =\tfrac{\pi }{2}$, *ε* = 1, *ϕ* = 0.1 and *φ* = 0°. A very interesting result can be observed from this figure. At low *S* values (0.1 ≤ *S* ≤ 0.3), Ag appears with higher enhancement in the heat transfer rate compared to other nanoparticles. However, by increasing the thickness of the porous layer, the Al_2_O_3_ nanoparticles tend to transport more heat within the cavity. In other words, adding Al_2_O_3_ nanoparticles to the water in a cavity with a higher porous layer thickness helps to transport more heat due to the lower thermal expansion of the Al_2_O_3_ nanoparticles.

## Conclusions

This work considers the problem of natural convection flow of a nanofluid in an inclined square enclosure with a partially-saturated porous layer with variable sinusoidal temperature on two opposing sidewalls based on Darcy’s law and the Boussinesq approximation. The finite difference methodology is utilized for solving the non-dimensional governing equations with the related boundary conditions. Detailed numerical data for the fluid flow and thermal distributions within the enclosure, and the local and average Nusselt numbers are exhibited graphically. The remarkable conclusions in the study are provided below:

1. A significant enhancement appears on the flow structure by applying lower inclination angle. This resulted in the appearance of the streamlines as a clockwise rotating cell within the nanofluid layer. In another words, imposing a lower inclination angle value is forced the streamlines to form a singular rotating cell which are similar to the constant temperature distribution.
2. The absolute lower values of the strength of flow circulation increase with the addition of solid volume fraction, while the significant reduction on the absolute higher values of the strength of the flow circulation appears for all nanoparticles types. This happens due to the viscosity forces and the inertial force changing.
3. The curved lines of the local Nusselt number on the left vertical wall are enhanced weakly by changing the phase deviation. Meanwhile, the heating domain on the right wall upwards and the cooling domain moves downwards which results in the local Nusselt number to form a sinusoidal shape caused by the phase deviation enhancement.
4. The rate of heat transfer is considerably influenced by the inclination angle increment affected by the velocity variation. The huge rise of the heat transfer is gained by changing the phase deviation. The increment and the reduction of average Nusselt number are indicated by the fixed inclination angle.
5. At low porous layer thickness, Ag appears with a higher enhancement in the heat transfer rate compared to other nanoparticles. However, by increasing the porous layer thickness, the Al_2_O_3_ nanoparticle tends to transport more heat within the cavity. This is due to the lower thermal expansion of the Al_2_O_3_ nanoparticle.
